# Non-Destructive Testing of the Longest Span Soil-Steel Bridge in Europe—Field Measurements and FEM Calculations

**DOI:** 10.3390/ma13163652

**Published:** 2020-08-18

**Authors:** Mikołaj Miśkiewicz, Bartosz Sobczyk, Pawel Tysiac

**Affiliations:** 1 Department of Mechanics of Materials and Structures, Faculty of Civil and Environmental Engineering, Gdansk University of Technology, 80-233 Gdańsk, Poland; mikolaj.miskiewicz@pg.edu.pl; 2 Department of Geodesy, Faculty of Civil and Environmental Engineering, Gdansk University of Technology, 80-233 Gdańsk, Poland; pawel.tysiac@pg.edu.pl

**Keywords:** non-destructive testing, soil-steel bridge, terrestrial laser scanning, finite element method (FEM), modelling and simulations

## Abstract

The article describes interdisciplinary and comprehensive non-destructive diagnostic tests of final bridge inspection and acceptance proposed for a soil-steel bridge made of corrugated sheets, being the European span length record holder (25.74 m). As an effect of an original concept a detailed and precise information about the structure short-term response was collected. Periodic diagnostics of bridge deformations was done one year after it was built. Load test design was based on numerical simulations performed by means of finite element method (FEM). In situ measurements were done with the aid of: inductive sensors, optical total station, and terrestrial laser scanner. The results produced by terrestrial laser scanning were used to build a precise image of structure deformation in 3D space during the tests. The accuracy of laser mapping was significantly increased using the information coming from total station and inductive sensors. These have higher accuracy and therefore can be used as reference. Thus, new quality in measurements is introduced. Good correspondence between in situ values and FEM estimations was achieved. Therefore, such a combination of testing methods can be used in non-destructive diagnostics of structures and is an interesting alternative for the standard approach, in which the measurements are done in limited number of points.

## 1. Introduction and Research Background

Engineering, mechanical, aerospace, offshore structures, etc., or their parts, elements, or connections are subjected to various and complex loading conditions during their life cycle. Sometimes these conditions are not properly identified during their design or, basically, could not have been predicted because of some accidental circumstances that emerge at the site. Consequently, failures and damages of the aforesaid objects occur. Because of that, great attention is focused on understanding of the structures response, enabling appropriate failure prevention, determination of real properties of the built-in material, identification and detection of damage, and failure mechanisms or monitoring of structure health (SHM). A lot of review papers have been written that gather knowledge about non-destructive testing of different structures, see for example [1,2,3,4,5,6]. In this article, we deal with non-destructive testing issues related to: detailed, automated, high-accuracy geodetic measurements, especially using terrestrial laser scanning (TLS); determination of structural response of a bridge; structural identification (St-Id) processes; and structural health monitoring (SHM). Therefore, recent achievements in this field are shown below.

In situ, field tests and measurements are commonly utilized for the purpose of non-destructive testing. In [7] TLS automatic inspection system, enabling detection and measurement of damage together with the verification of the quality and durability of surface repairs, as required by industry standards, is presented. A new approach for assessing the dimensional accuracy and structural performance of spatial structure elements, using three dimensional (3D) laser scanning, has been shown in [8]. In the paper [9], a hybrid digital reconstruction of a structure geometry using digital close range photogrammetry and laser scanning techniques is described. A comparison between the TLS measurements and the close-range photogrammetry done for deforming concrete beams, using the Structure from Motion algorithm, has been discussed in [10]. The article [11] reviews the abilities and drawbacks of a variety of remote sensing technologies, including laser scanning, and their applications for the purpose of automated measurements on a building site. The problems of structural identification by means of TLS and finite element method (FEM) are also considered. Some case studies in this field has been done for example for a historical minaret in [12] or mitre gates in [13]. Deformations of arched structures has been analyzed in [14] based on TLS measurement and FEM estimations, which were calibrated according to TLS results. Some other interesting case studies, in which TLS was employed and FEM calculations were done are available in [15,16,17]. Automated geodetic measurements are also used for the purpose of SHM. Some possibilities and case studies for timber structures are discussed in the review [1]. Laser scanning, refer to [18], has been used to investigate the health of historic masonry tower in Italy. Application of global position system (GPS)-based measurements of a cable stayed bridge in Romania, done for the purpose of SHM, is presented in the paper [19]. Dynamic responses of a suspension bridge in China have been monitored by means of high sampling-rate robotic total station, as it is described in [19]. Application of different sensors and geodetic measurements for the purpose of short and long term measurements of pedestrian bridge response, as well as a structural health monitoring (SHM) system to assess the behavior of a composite footbridge made of fiber-reinforced plastics (FRP) is discussed in [20]. Other example of composite bridge field tests is shown in [21]. An example of the use of interferometric radar to measure bridge deflections and evaluate its health is presented in [22]. Non-destructive testing of structures can also be done with the help of theoretical and computational models, numerical modelling, or simulations. These are used either independently, for example [23,24,25], or in combination with other testing methods, like the ones presented in preceding paragraphs. Some other examples of non-destructive testing supported by FEM are presented in the papers [26,27,28,29].

The above mentioned articles show that detailed, automated, high-accuracy geodetic measurements are important and are often employed for the purpose of non-destructive testing. They also point out high relevance of numerical models and computational techniques that support the testing. Nevertheless, the study of recent literature revealed a research gap related to the abilities of automated deformation measurements. The accuracy of TLS surveying, even when it is improved by the use of other devices or algorithms, is not better than 1 mm (refer for example to papers cited in the preceding paragraph or to [30,31,32,33]). There are structures experiencing very small displacements in typical conditions and therefore, automated geodetic measurements cannot be used in every case. An attempt to deal with this issue is discussed here.

In this article, we present interdisciplinary and comprehensive diagnostic, non-destructive tests of final inspection and acceptance of soil-steel single span bridge. Soil-steel bridges, viaducts, or culverts are structures built of flexible steel corrugated sheets or stiff concrete shells, which are covered with compacted soil having large angle of internal friction—backfill. Typically, span length of bridges of this type ranges from a few to several meters, rarely exceeding 25 m, while the backfill depth at the mid-span ranges usually from 0.5 m to a few meters (refer for example to [34]). The shell, buried in ground, is either a vault or a pipe [35]. Soil-steel bridges are designed in such a way that the constructional elements interact with each other to sustain their self-weights, weight of additional equipment, and traffic loads. The final effect of this interaction is beneficial. The bridges of this kind have the following advantages. They have specific architectural style. Their cost of construction is relatively low compared to the construction cost of steel or concrete bridges with standard abutments. This is because in the case of soil-steel bridges, the foundations are smaller, there are no abutments and the superstructure is made in the majority of the backfill. The time of the construction is short. Maintenance works for the soil-steel bridges are almost unnecessary. More information about this particular structural solution can be found in the handbook [35].

The analyzed bridge is a part of the national road number 16 linking the cities of Grudziądz and Olsztyn in Poland and is located near Ostróda city. To the best knowledge of the authors of this paper, this is the European span length record holder (25.74 m) in the category of soil-steel bridges. Because of that, it was very important to collect a lot of detailed information about the structure condition and its response under loads and when the bridge is unloaded. Therefore, standard programme of final inspection and acceptance, including mainly measurements of the bridge displacements in limited number of points under short-term static loads and its modal properties, was extended. In consequence, inter alia, detailed laser scanning of the bridge deformations was done, as well as a check of stresses in the steel shell.

TLS method was chosen to gather information about the bridge displacements, because it allows to capture image of the whole deformed shell in a short period of time. Nevertheless, preliminary FEM simulations of the short-term bridge response revealed that its elastic deformations under traffic loads are very small and do not exceed 2 mm. Therefore, in this case, in order to get reliable measurement results, the accuracy of TLS needed to be additionally improved. As reported in the literature review, currently, maximum resolution of TLS, even when it is supported by the use of other devices or algorithms is 1 mm and would have been insufficient.

In order to overcome this problem a new approach for high-accuracy scanning is proposed. We claim that TLS measurements can be enhanced using the information coming from total station and inductive sensors, that have much higher accuracy. Its application for the purpose of short-term static test surveying is the main goal of this research.

Additionally, deformations of the bridge, after one-year from the date the structure was built, were also checked by means of laser scanning. Moreover, computational model of the structure is created in the FEM environment in order to evaluate appropriateness of the bridge behavior and the measured values are compared with the estimated, calculated ones. The FEM model is calibrated using the measured data. Therefore, this paper is concentrated on the selection and presentation of appropriate non-destructive testing tools and methods, including FEM computational models, that enable to gather comprehensive and detailed information about the state and response of the analyzed soil-steel bridge structure. The main attention is focused on comprehensive, high-accuracy, geodetic measurements of bridge deformations and their comparisons with FEM response predictions.

## 2. The Soil-Steel Bridge

**Figure 1:**
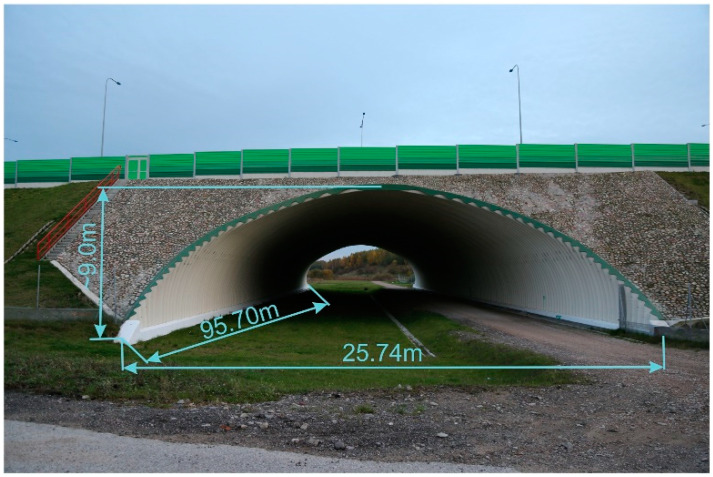
Soil-steel bridge side-view taken in October 2019.

**Figure 2:**
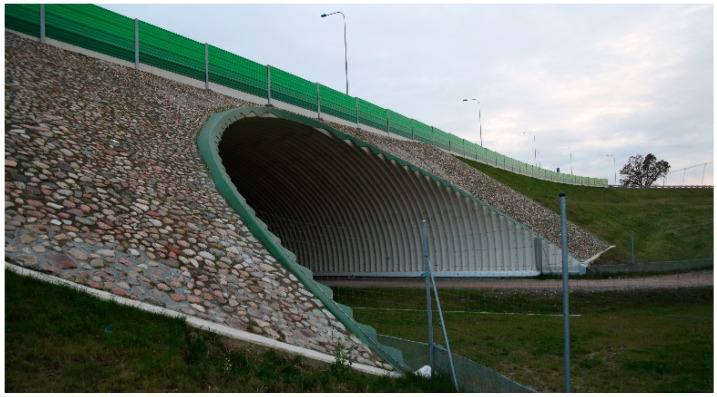
Close up of one of the bridge sides taken in October 2019.

**Figure 3:**
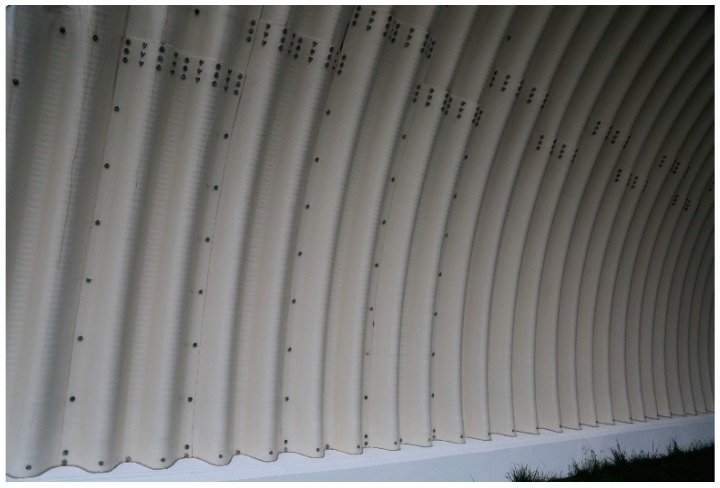
The corrugated steel sheets and their bolt connections.

The considered soil-steel bridge is a new one, built in 2017. It is a single-span arch structure, as shown in Figure 1 and Figure 2. The steel shell is made from UltraCor, 9.5 mm thick, corrugated sheets, produced by ViaCon Polska Ltd. company ([36]) (Rydzyna near Leszno, Poland), which are shown in Figure 3. The corrugated sheets are connected to each other with bolts. S315MC steel was used to produce the shell. The bridge foundations are built of Franki piles. The UltraCor structure is covered by soil compacted in a way that its relative density is not less than 98% of the maximum density determined in the laboratory. In the close vicinity of steel sheets, the compaction is not less than 95%. The steel arc span length is 25.74 m, whereas the total length of the bridge equals 95.70 m. The arc sagitta equals 9.0 m. These basic dimension, characterizing the structure, are depicted in Figure 1.

The bridge carries the national road number 16 and crosses a path for animals and a road built for the purpose of road maintenance (refer to Figure 1).

It is worth to mention that due to the aforesaid dimensions (large total length) it may not be so obvious if this structure should be named as a bridge or a tunnel. Nevertheless, we claim that this is a bridge from the following reasons. The soil-steel structure is made of flexible corrugated sheets and there is no base slab below the service road. Moreover, according to the polish law, appropriate road signs need to be put at the tunnel entrance and its exit. As seen in Figure 1 and Figure 2, there are no such signs. In addition, under the terms of the construction contract the structure was named as a bridge, which meets the definitions provided in the regulations.

## 3. Non-Destructive Testing Program

**Figure 4:**
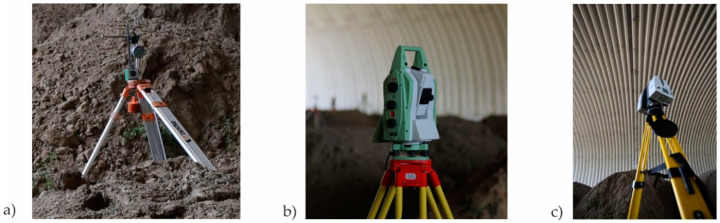
Devices and sensors used to measure basic bridge properties: (**a**) vertical translation inductive sensor, (**b**) total station, (**c**) laser scanner.

Each bridge, according to the Polish Regulations and instructions (refer to [37]) has to be tested before it is accepted for exploitation, to decide whether it is built appropriately or not. The final acceptance and inspection of the considered one took place on 18 July 2017. It was carried out by Aspekt Laboratorium Ltd. (Jaworzno, Poland) and a Research team from Gdańsk University of Technology (GUT) Department of Mechanics of Materials and Structures and Department of Geodesy. In consequence, basic parameters were checked for the bridge following the instruction [37]. The stiffness of the bridge was established based on the measurements of vertical deflections of the structure under static loads. This was done by means of inductive sensors and total station surveying (see Figure 4). The stability of supports was checked through the foundation settlements measure, carried out using precise geodetic levelling. Modal properties were determined using impact hammer, inductive displacement sensors and accelerometers. Visual inspection of the bridge was done after series of tests, in order to check if the structure sustained any damage, cracking, breakage, etc. The bridge passed all the standard tests.

In addition to standard measurements described in the previous paragraph, extended test programme was launched and accepted by the Road Authorities, Contractor, and Designer, because of large dimensions: length, width, and height of the steel shell, which make the bridge the current European span-length record holder in the category of soil-steel road bridges. Therefore, additional dynamic tests were done to identify the eigenmodes and eigenfrequencies of the structure with higher precision. Also terrestrial laser scanning of steel shell deformations was done (see Figure 4) in order to capture short-term deformations of the bridge and perform its periodic diagnostics.

**Figure 5:**
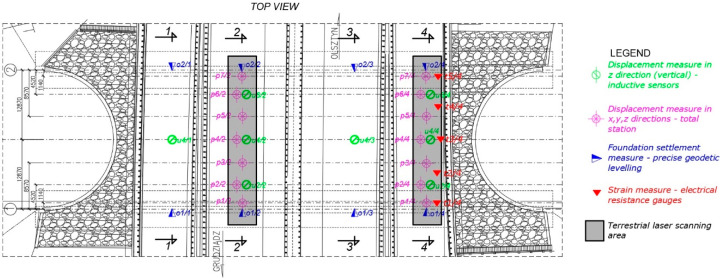
Location of the network of sensor used to collect the data during the static tests. Top view of the bridge.

**Figure 6:**
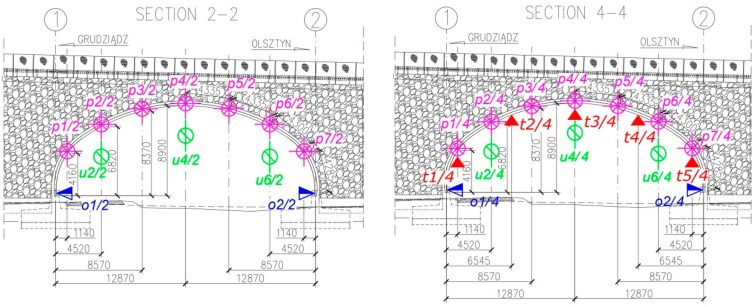
Location of the network of sensor used to collect the data during the static tests. sections 2-2 and 4-4 (dimensions in (mm)).

In this case a novel approach to increase the quality and accuracy of the scan has been proposed by the authors of this paper and it was successfully achieved during the tests. It is described in details in the next chapter. Finally, strains of the steel corrugated shell were monitored at the site during the tests using electrical resistance strain gauges and corresponding stresses were calculated. It needs to be emphasized that such an approach to non-destructive testing during final acceptance and inspection of a bridge is rarely seen in diagnostics of the structure. It enabled to collect a very detailed information about the bridge. Location of the network of sensor used to collect the data during the static tests is depicted in Figure 5 and Figure 6. In Figure 6 only sections 2-2 and 4-4 are shown. This is because of the fact that the loads were applied to the bridge on the road lanes above these sections and in sections 1-1 and 3-3 the deformations were rather small. What is more terrestrial laser scanning of the short-term bridge deformations, being one of the most important aspect of this research, was done in sections 2-2 and 4-4.

**Figure 7:**
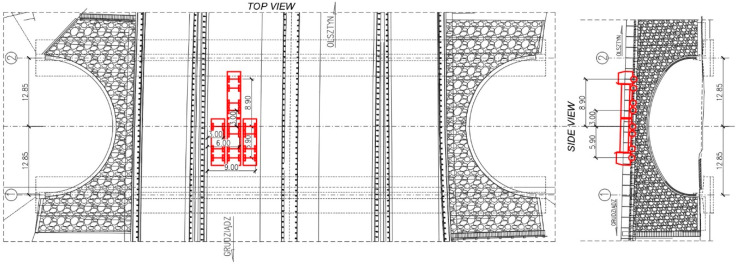
Schematic drawing of trucks locations during the S1 test configuration (dimensions in (m)).

**Figure 8:**
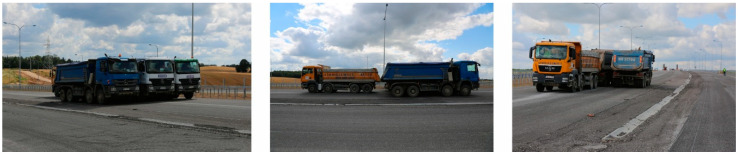
The trucks, positioned during the S1 test configuration. Views from three different perspectives.

To load the bridge and study its response during the tests 4-axle trucks were used. The total mass of each of the truck was 32 tons. The trucks were positioned in a way to maximize the loading effects in the measurement points. Two configurations of trucks positions were studied during the classical static tests. These were denoted as S1 and S2. The trucks were put on the bridge one by one, steadily in columns. Schematic drawing of trucks locations during the S1 test configuration is presented in Figure 7. Views from three different perspectives that document trucks positions during the S1 test configuration are shown in Figure 8.

**Figure 9:**
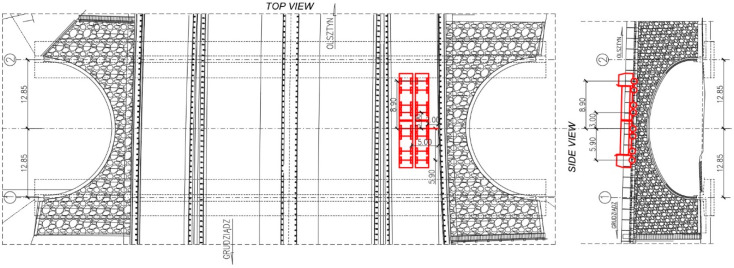
Schematic drawing of trucks locations during the S2 test configuration (dimensions in (m)).

**Figure 10:**
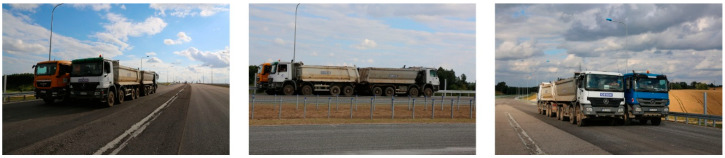
The trucks, positioned during the S2 test configuration. Views from three different perspectives.

Schematic drawing of trucks locations during the S2 test configuration is presented in Figure 9. Views from three different perspectives that present trucks positions during the S2 test are depicted in Figure 10.

**Figure 11:**
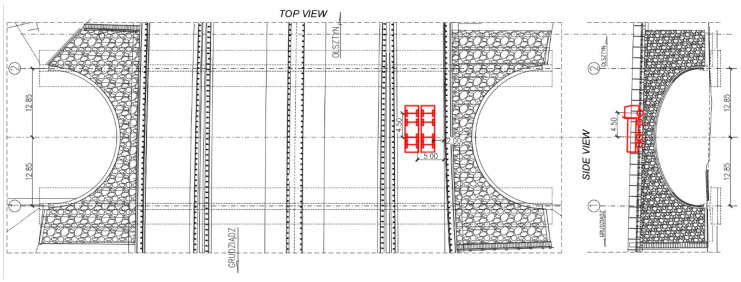
Schematic drawing of trucks locations at M24 stop (dimensions in (m)).

**Figure 12:**
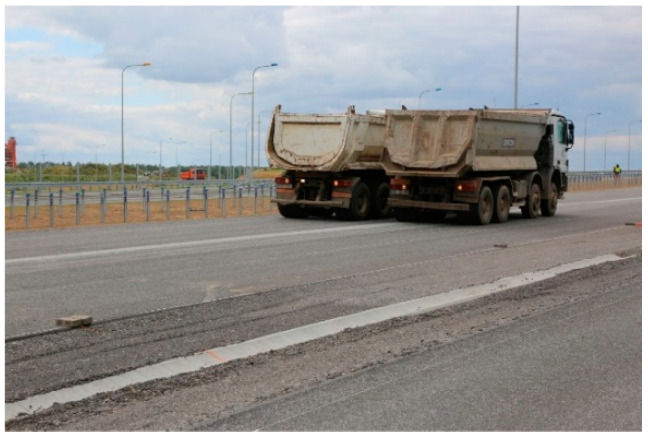
The trucks moving between M23 and M24 testing positions.

The bridge response was also checked under moving ballast of two trucks positioned side by side. These test configurations were denoted as M11 to M16 for the trucks standing on the road lane above 2-2 section and M21 to M26 for the trucks located above the 4-4 section. The trucks stopped in 6 equally spaced locations over the bridge. In Figure 11 schematic drawing of trucks locations at M24 stop is shown, whereas trucks moving between M23 and M24 testing positions are depicted in Figure 12.

More details about the final acceptance tests, carried out in Poland, can be also found in [38,39,40]. It is worth to mention that the concept of final acceptance tests based on moving loads is often considered (refer for example to [41,42]). Soil-steel bridges are structures, which response strongly depend on the behavior of the backfill. When the bridge is loaded the backfill and the shell deform together and thus its shape can slightly change. This deformation will be stronger when a flexible shell is used to cross the obstacle. When moving load concept testing is done and a bridge is loaded in a sequence of steps, the change of its shape should be much smaller, as the whole structure can easily accommodate the loads and the permanent deformations do not occur. In such a case the interpretation of measured results is easier. On the other hand, a moving load is smaller than the extreme load that can be applied to the structure. From this reason both types of load application, namely in the classic static way and with moving trucks, are important in the case of this bridge.

## 4. High Quality Laser Scanning—Methodology and Short-Term Static Tests Results

The preliminary numerical simulations of the structure response revealed that the shell deformations under standard loading conditions are very small—a few millimeters only. Therefore, we tried to find a method allowing us to gather as much data, about the bridge behavior, as it is possible in a relatively short period of time, having at the same time very high quality and accuracy. Owing to the research gap, identified in the introduction, we came up with an idea to perform TLS surveying of the bridge cross sections. Then, adjust and transform the scanning results appropriately, based on total station and displacement inductive sensor measurements, to significantly increase its accuracy. It is worth mentioning that soil-steel or concrete-soil composite bridges are typically subjected to in situ tests in a limited number of points and using some basic techniques of measures (refer to papers [43,44]). Some more advanced studies can be found in [41,42,45,46,47,48].

If the problem is defined as stated above, the in situ measurements methodology is divided into two stages. A properly prepared testing programme is launched at first, including laser scanning, total station and inductive sensors displacement measurements. During the second stage, proper post-processing of the data is done. Our experience in laser scanning measurements post-processing and aggregation of the results is available also in the following publications [49,50,51,52]. Our recent use of laser scanning during the final acceptance test is described in [53]. The measurements were done for the new European record holder in span length among extradosed type bridges.

The procedure and methodology for obtaining precise and accurate high quality results of terrestrial laser scanning is now presented. Leica P30 LiDAR (Light Detection and Ranging) laser scanning technology and robotic Leica TM50 total station were used for the purpose of geodetic surveying. It is worth mentioning that in this case the accuracy of the point cloud is the main research problem. According to the manufacturer’s specifications, the accuracy of a point model, created during a single scan operation in one position, is about 1 mm and is going to be improved later on.

At first, appropriate preparation of the test field was done. The laser scanner was positioned in the line of the axis of each measured bridge cross sections 2-2 and 4-4 (as shown in Figure 5) and remained in the same place during each testing. Hence, the alignment of scan positions was not an issue. The total station was placed in a way that all the target prisms in the measurement points were visible. The positions of prisms (total station measure) and targets (laser scanning) were in accordance with the testing project. It has to be mentioned here, that during the tests a decision was made to do the total station survey in 9 instead of 7 points. Additional measures were done in o1 and o2 points. Thus, for each cross section under consideration, regarding the total station surveying, data were gathered from o1, o2, p1, p2, p3, p4, p5, p6, and p7. Moreover, two reference points, which are not marked in Figure 5, were established on the steel shell in the region where the deformations under the loads were very small, but still in the vicinity of the measured section. These were used to check the correctness of deformation registration. Additionally, the appropriateness of the total station surveying was checked using the data gathered by inductive displacement sensors. In Table 1 vertical displacements registered by total station (p2/4, p4/4, p6/4) and inductive sensors (u2/4, u4/4, u6/4), during S2 test, when maximum load was put on the road lane right above 4-4 section, are compared.

On the basis of the results presented in Table 1 and owing to the precision of the used devices (inductive sensor ±0.01 mm, total station ±0.1 mm), it can be concluded that the total station measurements were appropriately done. It is worth mentioning that when a total station measurement is uncertain, additional correction of the vertical displacement can be done with regard to the inductive sensor measure. Consequently, such an updated (adjusted) result is used later on, during the process of TLS shape determination of the deformed bridge. However, in this case it was not necessary to do any additional adjustments.

The maximum distance of the targets from the scanner was approximately 20 m and the time of measurement of 9 points by the total station was about 3 min. Therefore, the laser scanning resolution was set to 3 mm per 10 m. In consequence, each scan of the whole bridge cross-section deformations lasted 3 min and 30 s. This allowed for obtaining the best measurement resolution and relatively many points of measure that were used at a later stage to create a curve representing the deformed shape of the structure. A large number of data, which was saved in such a case was not an issue, because the used instrument has the ability to reduce the noise at the level of 0.4 mm per 10 m. The surveying was done for the unloaded and loaded structure, according to the final acceptance testing programme.

After the survey ended, the post-processing of the geodetic data began. The data projection was used to compare the results from laser scanner with the ones from the total stations. In order to do so and because the total station measurements were taken in 9 points only, interpolation of the total station results between these points was done using the polynomial function. Similar considerations can be found in [54]. The chosen polynomial was of 9th degree. Thus, approximated shape of the tested cross section based on total station survey has been determined. The fitting root mean squared error (RMSE) came out at about 2 mm in all cases. This is compatible with measuring capabilities of the scanner. Therefore, we can say that the interpolation is accurate.

After that, approximation was done by means of the least squares methods of the laser scanning point cloud in order to find its best fitting with regard to the polynomial function resulting from the total station measurements. In effect a precise image of the bridge cross section in the reference state was obtained. It should be mentioned here that appropriate transformation of coordinates obtained from the total station into the coordinate system of laser scanner was carried out as well.

**Figure 13:**
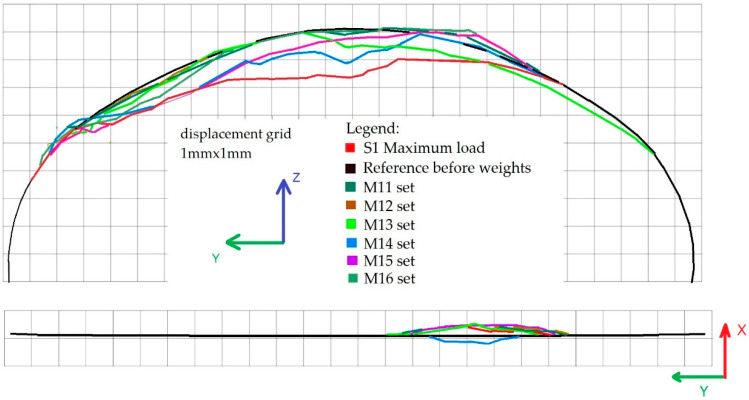
Deformations of the bridge cross section 2-2 in the Y-Z (top) and X-Y (bottom) planes under loading schemes S1 and M11–M16.

**Figure 14:**
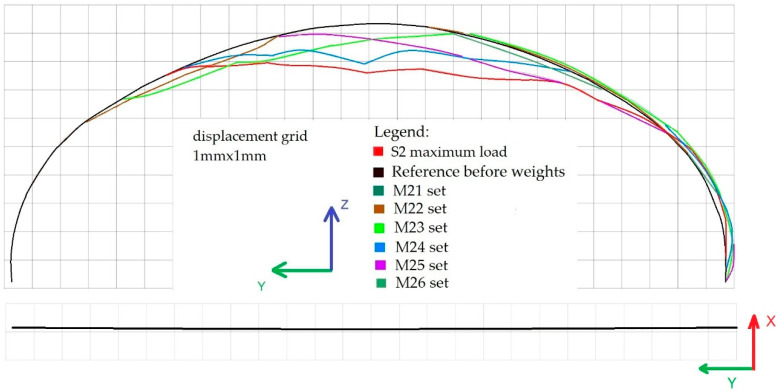
Deformations of the bridge cross section 4-4 in the Y-Z (top) and X-Y (bottom) planes under loading schemes S2 and M21–M26.

At that point, data were collected, containing images of reference and deformed states of the structure. Therefore the displacements of the steel shell were easily calculated for all the cross sections under consideration. The resulting deformations of the bridge cross section 2-2 in the Y-Z and X-Y planes under loading schemes S1 and M11–M16 are shown in Figure 13. Similarly in Figure 14, shell deformations are shown for the 4-4 cross section for the following loading conditions S2 and M21–M26.

It should be noted that temperature changes may influence response of the bridge. However, the weather, on the day of tests, was cloudy or partly cloudy (see Figure 8, Figure 10 and Figure 11) and therefore the bridge was not strongly exposed to the sun and maintained constant temperature. For this reason discussion about deformations of the bridge due to temperature changes can be omitted.

In the end, we did manage to adjust the resolution of the laser scanning to the level of ~0.1 mm in lateral X and Y directions (in the location of total station prisms) and ~0.01 mm in the vertical Z direction (where inductive sensors were located) for the 2 mm × 2 mm grid of measurement points. The resulting accuracy of the laser scanning was ~0.3 mm, while the standard one obtained without any improvements is ~1 mm, as mentioned before. It is worth reminding, that in order to attain the accuracy, we adjusted results coming from laser scanner in the studied cross sections based on the information provided by the remaining sensors. Consequently, every point, registered by the scanning was influenced by this operation and therefore, accuracy of the whole scan was improved. Finally, it was possible to benefit from one of the advantages of the scanner which is its high scan rate in comparison to the other methods used, still maintaining accuracy of the gathered data. Therefore, we proved that the laser scanning technology can be used in measurements of displacements, being less than 1 mm. We consider our solution as original and innovative because such an aggregation of data coming out from different surveying devices and appropriate interpretation of results has not been done before in the field of static measurements of soil-steel bridges. Finally, we can say that we found a method to control and maintain very high resolution and accuracy of terrestrial laser scanning. Thus, very detailed images of the bridge cross sectional deformations in 3D were captured. It is also important, that the image of the unloaded bridge satisfies the conditions of the reference state in the case of structure monitoring and possible periodic diagnostics during its lifecycle. From the aforesaid reasons, we propose to include scanning of this type as a possible method of measurements during final acceptance and testing of bridge structures.

## 5. Periodic Diagnostics of the Bridge One Year after It Was Built

The next part of the research was focused on the periodic diagnostics of the bridge. In 2017 a scan of the whole unloaded steel shell had been made, which was repeated one year later. This enabled to check not only short-term elastic displacements, caused by real traffic conditions during final-acceptance and inspection, but also the eventual permanent displacement of the analyzed soil-steel structure. The periodic diagnostics is done to learn about the order of the bridge permanent displacements, whose occurrence is typical for this type of structures, during their exploitation. Here, great accuracy of measurements is not so important, as we expect that the displacements, that could have occurred, can be as big as a dozen or so mm. Therefore, additional improvement of scanning accuracy, as done for the purpose of short-term tests, presented in the Section 4, is not done.

In 2017, after short-term static tests were completed, the bridge was unloaded and scanning control points were stabilized in order to prepare for additional measurements. After that, full image of the bridge was registered. Because of the large dimensions of the structure, position of the scanner during the measurement process was changed 12 times. This process was repeated in the year 2018.

It needs to be emphasized that the surveying in 2018 was done at weather conditions similar to the ones observed the year before. Therefore, the results of periodic diagnostics are not affected by temperature variations.

**Figure 15:**
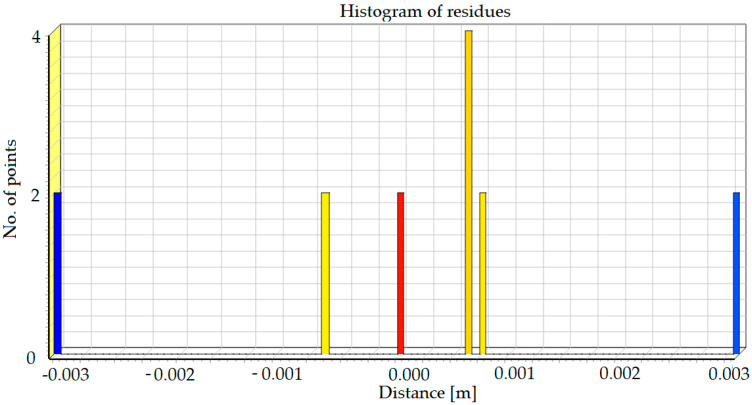
Histogram of residues which shows the precision of scan alignment.

As new scanning required new alignment of the device, the TLS accuracy could have been additionally checked. The Iterative Closest Points (ICP) algorithm was used to align scan positions correctly, besides of reference points that were stabilized outside of the structure. The positions of elements, which were used for the algorithm processing, were not changed during this time period and they have not been influenced by any external factors. The results of this alignment are presented in the form of histogram in Figure 15.

The standard deviation resulting from the alignment was *σ* = 1.6 mm. Therefore, in order to reliably estimate the accuracy of the measurement, in accordance with the principles of normal distribution, we assumed that it is 3 × *σ* = ~5 mm. In consequence, the accuracy of computed deformation differences (registered in the period between 2017–2018) should have not exceeded this value.

As usual, the noise reduction was done during the process of point cloud data filtration. The noise reduction algorithm described in [55] was used here, which locally fits a surface into the point cloud and then remove points that are too far from that surface. In the end, it was possible to exclude points lying outside the possible deformation shape that had a negative impact on the final results and to retain only the ones maintaining reasonable accuracy.

**Figure 16:**
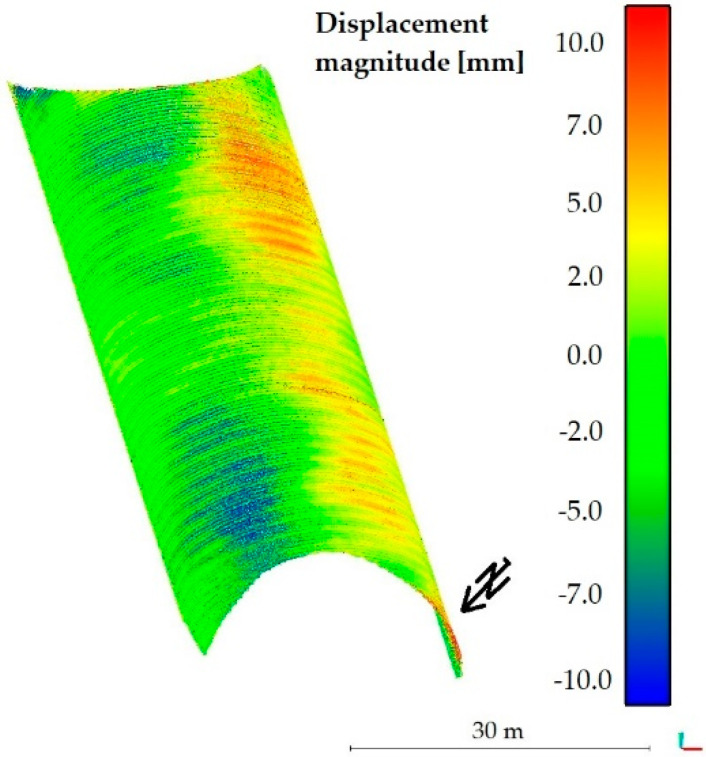
Periodic diagnostics of bridge deformations (displacement magnitude) measured one year after the final acceptance and inspection.

After the noise reduction had been done, it was possible to calculate the periodic diagnostics displacements of the steel shell that occurred between the years 2017 and 2018. The method described in paper [56] (available in CloudCompare software) was used for this purpose. This particular approach was chosen because most of the available comparative methods are based on the determination of the closest distance from a point or require 3D modelling of the compared surface. Thereby, they eliminate the problem of roughness, which cannot be eliminated here, as the corrugation of the steel sheets is an important feature of the whole structure and cannot be omitted. To find the distances between two point clouds, two main steps were done. First, normal surface and its orientation in 3D was estimated on a scale consistent with local surface roughness. Then, the mean surface change was measured along the surface normal direction and the local confidence interval was determined. This, in our opinion, allowed to obtain reliable information about the structure, which has varying roughness, as evidenced by the observed noise in the cloud of points and terrain diversity. The roughness is also reflected by the average change along the surface normal direction, as it was done in the second major calculation step of this procedure. In effect the distances between two point clouds were obtained and the deformations contours (magnitudes of displacements) are shown in Figure 16.

**Figure 17:**
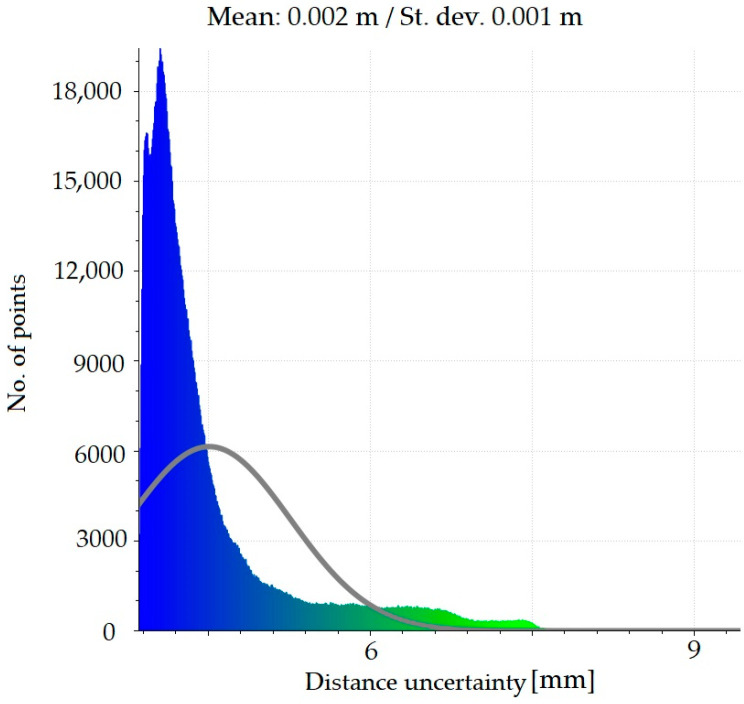
Distance uncertainty statistic.

The results of the calculations presented in Figure 16 reveal that the upper part of the bridge has moved to the south up to 7 mm, while the shell in the vicinity of foundation remained undeformed. To check the accuracy of the obtained deformations a series of statistical tests, based on the Gaussian normal distribution, were run. Figure 17 presents the statistics of the Gauss function corresponding to the calculated distance uncertainties. On this basis it can be stated that the results were correctly determined in relation to the reference shown in Figure 15.

Owing to the above, the uncertainty of the measured deformations (Figure 16) equals approximately 2 mm (within the computed *σ* from alignment). This means that the laser scanning enabled to register all of the points characterizing the largest geometric changes. Nevertheless, this uncertainty suggests that some discrepancies of the measurements occurred during the scanning. These resulted possibly from the standard issues connected with stabilization of points, their indication, and establishment. However, because of the fact that the measurements were repeated one year after the final acceptance tests and during this period of time the construction works were still ongoing, such an accuracy of measurements is to a large extent satisfactory. For example in paper [51] laser scans of an external system were done using other techniques and the resulting precision and accuracy was not as good as here.

**Figure 18:**
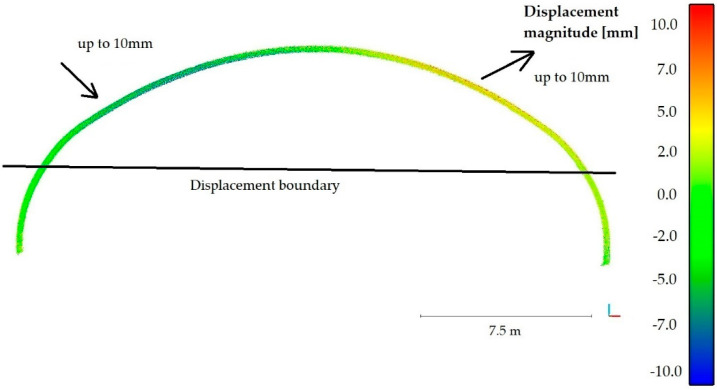
Displacements of the unloaded steel shell 2-2 section one year after the final acceptance and inspection tests.

**Figure 19:**
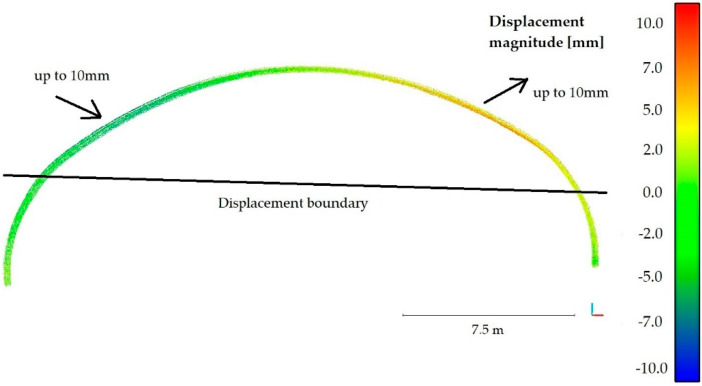
Displacements of the unloaded steel shell 4-4 section one year after the final acceptance and inspection tests.

Periodic diagnostic deformation images were created also in sections 2-2 and 4-4, that were previously tested during static tests in 2017. This enabled to additionally cross-check calculations of deformations of the whole bridge, presented in Figure 16. To create them it was required to perform a number of computational operations, enabling interpretation of deformation changes of the bridge. The results obtained from scanning during short-term tests, were quite helpful in achieving this goal. Displacements of the unloaded steel shell one year after the final acceptance and inspection tests is shown in Figure 18 for the 2-2 section and in Figure 19 for the 4-4 section. The directions of the extreme values of displacements are also shown in Figure 18 and Figure 19.

The aforesaid results reveal, that during one-year period of time, the bridge did not exhibit deformations below the black border line, marked in the figures and named as “displacement boundary”. In view of that, we noticed a certain negative feature of the computed periodic diagnostics deformations (see Figure 16, Figure 18 and Figure 19). Despite attaining satisfactory result of calculations, the displacements were not obtained in the lower parts of the steel shell. However, because of the measurement accuracy, their exact values are not so certain and may rather indicate the displacement tendency. Similarly, this could have resulted from the properties of the algorithm used. On the other hand, what also is interesting, the “displacement boundary” intersects the shell approximately at the same height at which bolt connections were done (refer to Figure 3). As one year passed since the date of final acceptance tests, slippage of this connections could have occurred and some rotations of the shell have become possible in this areas. This may explain why displacements of the upper part of the shell are only observed. Hence, a research gap has been identified here. These aspects will be analyzed and addressed in further studies.

Finally, it can be concluded that the periodic diagnostics displacements measured one year after the final acceptance tests are relatively small, 7 mm ± 2 mm. Here, we would like to emphasize that the extreme values of permanent displacements are observed above the aforesaid “displacement boundary.” Therefore, maximum and certain displacement of the bridge did not exceed 9 mm. This is a very important information as the order of the permanent displacement was identified. Occurrence of deformation of this order is typical for soil-steel structures. Thus, the bridge experienced a change of its shape, which however does not affect its response and load-bearing capacity.

## 6. Numerical Analyses and Comparison of Results

### 6.1. Computational Model

In order to design the final acceptance test programme, the bridge response needs to be estimated. Therefore, numerical simulations of its behavior under static loads and natural frequency extraction were performed by means of FEM (refer to [57]) using Abaqus 6.14-2 code. The computational model was created according to the bridge technical drawings and it is a particular one. It is created to describe the global response of the soil-steel bridge and to check whether the assumptions and approach to design were appropriate. Thus, the computational domain needs to remain in correspondence with the precision required by the design process. From this reason, we employ basic modelling techniques and some simplifications. The model should not be overdetailed, but it still has to allow for obtaining reliable results.

**Figure 20:**
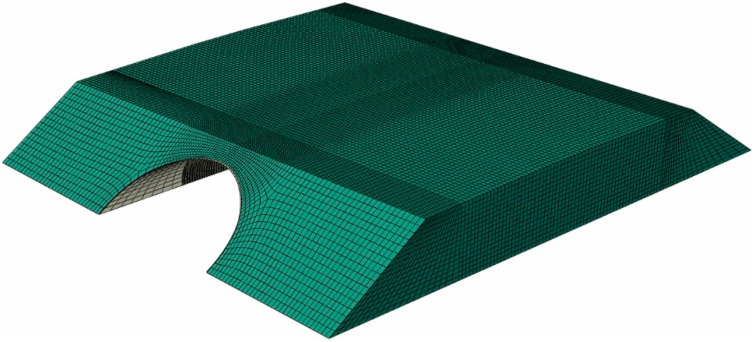
Finite element method (FEM) computational domain of the bridge.

**Figure 21:**
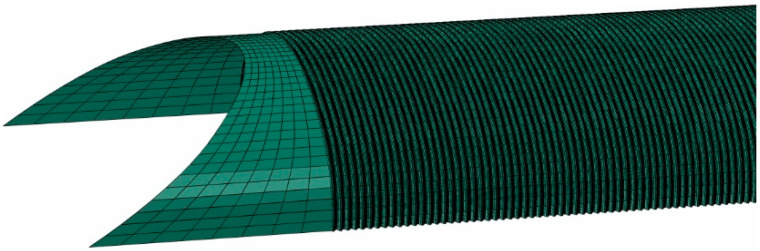
Steel shell in the numerical model: corrugated sheets in the center, equivalent shell close to the bridge ends.

Its size (distance from the bridge to the outer boundaries of the domain) was selected in such a way that it did not affect the results in the area of the steel shell. A structured mesh of finite elements was created. First-order solid C3D8I elements (refer to [58]) were used to model the soil continuum. These elements are enhanced by incompatible modes. This formulation enables very strong reduction of the locking effect, as stiffening in bending, being a consequence of the parasitic shear stresses or Poisson’s effect, is almost eliminated. The steel parts were represented by shell S4 elements (see for example papers [34,59,60,61] for other applications of shell elements) with linear shape functions and full 2 × 2 in-plane Gauss-Legendre integration scheme and some additional procedures preventing the locking effect. In the central part of the structure, namely in the region where the in situ measurements were taken, the corrugated sheets were modelled in detail having regard to the real geometry. A simplification was made close to the structure ends, where the steel sheets were treated as a shell endowed with equivalent orthotropic properties and thickness. It is worth mentioning that, often, the whole steel shell is treated as the one having some equivalent properties and uniform thickness (see for example papers [42,62]). Nevertheless, such a simplification cannot be done here, because we are interested in accurate estimation of stresses in the shell-corrugated sheets. The whole computational domain of the soil-steel composite structure is shown in Figure 20 and the detail depicting modelling approach of steel shell in Figure 21.

The soil and steel were treated in the analyses as isotropic homogenous materials. The elastic constants of the soil were determined on the basis of the requirement of backfill compaction—minimal relative density of the material should be not less than 98% of the maximum density of the soil achieved in the laboratory. This corresponds, according to the polish design standards still used by site engineers [63], to the elastic modulus being 170 MPa. However, often, at the site, the properties of the backfill are better than minimums. So it was also in this case. Aggregate with very good parameters was used to construct the backfill. The reports on the compaction acceptance of the backfill layers showed that the compaction was ranging from 100% to 103%. Therefore, other possible soil properties are also considered, with elastic modulus being 200 MPa (corresponding to 100% compaction) or 230 MPa (corresponding to 103% compaction). Thus, we try to calibrate the numerical model, based on the obtained measurements and assign soil properties enabling best fitting of the FEM estimations. The material properties of steel and soil used in the computational analyses are collected in Table 2. An evident simplification of the material law for the backfill is done. Nevertheless, it is justified by the following reasons. A global model is created and we focus mainly on the appropriate estimation of displacements and stresses in the steel shell. Therefore, detailed description of soil material law is not so important. The backfill main properties are calculated based on well-established engineering procedures, which should enable reasonable description of its response. Second, behavior of the bridge under design loads is recreated and by assumption this loading condition should not lead to any failure. What is more, if failure occur in the backfill, the real response of the bridge would be much different from the FEM estimation, which would also clearly indicate that the simplified approach is not a proper one. This will be verified in the next chapter. It is also worth mentioning that the backfill is treated in a similar way by others and good results are obtained, as presented for example in [64].

It also needs to be noted that the bridge was assembled in summer and its temperature during the tests was similar to the one at which it was mounted. There was only a couple of degrees difference. What is more, soil elastic modulus is only slightly affected by the temperature changes, as reported in [65]. Therefore, the material properties of the backfill does not need to be adjusted.

The following boundary conditions were assigned to the model. The steel shell is fixed in the foundations. Vertical displacements are restrained at the lateral lower surface of the embankment. The displacements perpendicular to the vertical side surfaces of the embankment are constrained as well. A contact was defined between steel shell and the surrounding backfill.

The loads were applied to the model in accordance with trucks positions, described in the Section 3 and their axle loads. In consequence, uniformly distributed loads were defined at truck tires-road contact area. The shapes of tire contact patches were simplified and treated as rectangular. No additional application of temperature loading was necessary to compensate eventual change of the bridge geometry due to the change of temperature during different testing configurations. This is due to the fact, described earlier as well, that the weather during the whole testing day (cloudy or partly cloudy) was basically the same, the bridge was not exposed to the sun, therefore variation of its temperature was negligible.

Finally, static analyses were executed to estimate bridge response during the in situ tests.

### 6.2. Comparison of FEM Estimations and In Situ Measured Values

**Figure 22:**
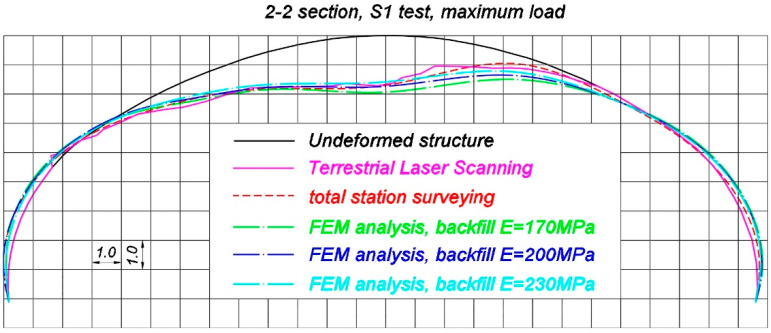
Comparison of the deformed shapes of the bridge in 2-2 section during the S1 test, when maximum load was applied, measured using terrestrial laser scanning, total station, and estimated by means of FEM (deformations in (mm) are 1000 times scaled).

**Figure 23:**
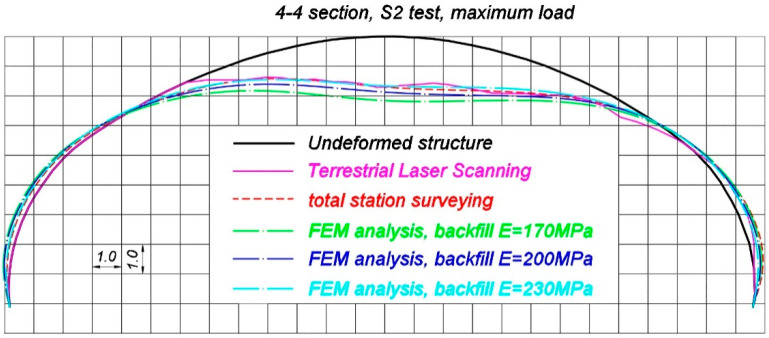
Comparison of the deformed shapes of the bridge in 4-4 section during the S2 test, when maximum load was applied, measured using terrestrial laser scanning, total station, and estimated by means of FEM (deformations in (mm) are 1000 times scaled).

**Figure 24:**
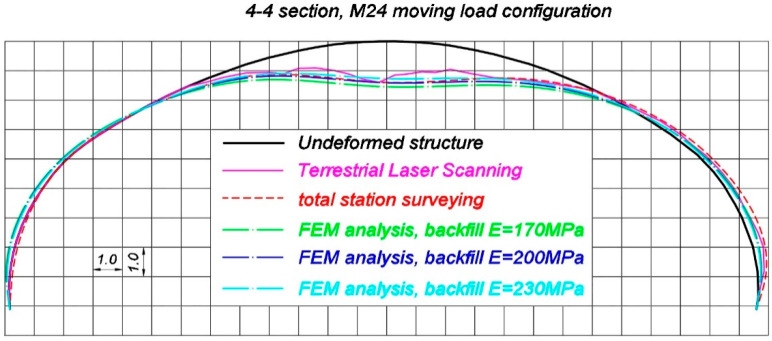
Comparison of the deformed shapes of the bridge in 4-4 section during the moving load M24 configuration, measured using terrestrial laser scanning, total station, and estimated by means of FEM (deformations in (mm) are 1000 times scaled).

Now comparisons of selected, representative results of the in situ measurements and corresponding FEM estimations are presented. Total deformations of the steel shell, measured by terrestrial laser scanning and total station are compared with the results coming from FEM calculations, in which different soil properties were assigned (refer to Table 2). These are shown in Figure 22, Figure 23 and Figure 24, correspondingly, in 2-2 section under maximum load during S1 test, in 4-4 section under maximum load during S2 test and in 4-4 section during M24 moving load test configuration. It is worth mentioning that typically elastic displacements are analyzed during final acceptance tests. However, this is a specific case of a soil-steel structure. When the bridge was unloaded after S1 and S2 tests a slight change of shell shape was observed. Since point-cloud is created as a result of laser scanning, it is very hard to calculate elastic deformations coming from TLS. This is the reason why total deformations are compared in this chapter. Nevertheless in the case of moving load tests (refer for instance to Figure 24) the total deformation is nearly the same as the elastic deformation of the structure, because such a testing approach allows to significantly reduce this effect, as it was described in the Section 3. It has to be additionally noted that total station surveying was done in 9 points, as shown in Figure 6, in each cross section under consideration. In Figure 22, Figure 23 and Figure 24, spline function was used to connect these 9 points and in effect deformed shape resulting from total station measurements was approximated.

It is seen in Figure 22, Figure 23 and Figure 24 that the FEM estimations, even for different variants of the backfill elastic modulus, are generally in accordance with the measurements with regard to their quality and quantity. The backfill elastic modulus is an important parameter that affect the bridge response. It is a bit underestimated, when E = 170 MPa. Better quality results are obtained for E = 200 MPa and E = 230 MPa. In order to investigate displacements of the steel shell in detail, now, the extreme vertical displacements, that were obtained in the p4/2 point for the test S1, p4/4 for the test S2 and the corresponding FEM values (for different backfill material properties) are analyzed in Table 3. In Table 3 both total (U_in-situ,tot_) and elastic (U_in-situ,el_) in situ measured displacements are presented to highlight the issue of the shell shape change when the bridge was unloaded.

In Table 4 vertical in situ displacements in the p4/4 point during the moving load tests M21–M26 are compared with the ones resulting from FEM calculations. Total displacement and the elastic values are not distinguished in Table 4, since they are almost the same.

The results from Table 3 and Table 4 reveal that the response of the soil-steel composite bridge is underestimated, when the backfill elastic modulus is E = 170 MPa. In this case, the elastic displacement ratios U_in-situ,el_/U_FEM_^E = 170^ for p4/2 and p4/4 locations, during S1 and S2 tests, are correspondingly 79% and 77%. The ratio between the biggest in situ vertical displacement during moving load test (M24 test configuration) and the corresponding FEM estimation U_in-situ,M24_/U_FEM,M24_^E = 170^ equals 84%. Similarly, for the backfill elastic modulus equaling 200 MPa, the ratios U_in-situ,el_/U_FEM_^E = 200^ in p4/2 and p4/4 points, during S1 and S2 tests are correspondingly 86% in both locations, while the ratio U_in-situ,M24_/U_FEM,M24_^E = 200^ for the M24 test configuration is 94%. Finally the results are compared for the backfill having assigned E = 230 MPa. In this situation the ratios U_in-situ,el_/U_FEM_^E = 230^ are 98% for p4/2 and 95% for p4/4 measurement point, whereas for the case of the moving load tests the ratio U_in-situ,M24_/U_FEM,M24_^E = 230^ equals 104%. Based on these comparisons, it can be concluded, that from an engineering point of view, the response of the bridge is well recreated for the backfill with E = 200 MPa and E = 230 MPa. As the moving load test is probably the most effective way to examine and measure the bridge response (this issue has been already described in Section 3), it can be stated the real properties of the backfill regarding its elastic modulus are somewhere between 200 MPa and 230 MPa.

Nevertheless, the geometry of the shell at the beginning of the tests was slightly different than it was designed, as it had adjusted its shape during backfilling. Moreover, it continued to adjust its shape during final acceptance tests under different loading conditions and the whole bridge experienced some small permanent deformations. This is typical for soil-steel structures. In consequence, during static and moving loads tests, the loads caused by trucks were applied to the bridge having a bit different geometry. Ideal geometry of the structure, based on the technical drawings of the bridge, was defined in the numerical model. This could have caused the differences between the compared in situ and FEM values shown in Figure 22, Figure 23 and Figure 24 and Table 3 and Table 4. It is also worth to mention that the bridge shell is built of small corrugated steel sheets which are connected to each other with bolts (as shown in Figure 3). Such a connection technique requires the steel sheets to overlap each other. In effect, the whole shell is somehow locally stiffened, which may contribute also to the global stiffness of the whole system. A continuous shell without connections is created in the computational model. Therefore, one may expect that the structure has an internal margin of safety. Owing to the aforesaid considerations, we claim that the backfill properties are close to the ones represented by elastic modulus which is 200 MPa. In effect, we can say that the computational model has been calibrated with aid of the measurements. Thus, it reflects the real response of the analyzed structure. In effect it can be used for the purpose of the bridge diagnostics or support interpretation of SHM data.

Finally, the stresses calculated on the basis of strains, registered during the static tests S2 in t2/4 and t4/4 points, are shown together with corresponding FEM predictions in Table 5. The results presented in Table 5 were calculated assuming that the elastic modulus of the backfill is E = 200 MPa, which has been calibrated based on the displacement measurements, being the most reliable ones in this research. The points t2/4 and t4/4 were located approximately in the quarter-spans of the steel arch and extreme values were obtained there. Additionally, stresses in t3/4 point are also compared in Table 5. In Table 6 stresses calculated from strains in t2/4 point during the moving load tests M21–M26 are compared with the ones resulting from FEM calculations. Total and elastic values are not distinguished in Table 6, since they are almost the same.

Similarly, as in case of displacements analysis, the stress ratios S_in-situ,el_/S_FEM_ for t2/4 and t4/4 from Table 5 are calculated and they are correspondingly 92% and 96%. The ratio between the biggest in situ stress during the moving load test (M24 test configuration) and the corresponding FEM estimation S_in-situ,M24_/S_FEM,M24_ is 88%. It has to be emphasized here, that because of characteristics of the used devices (strain gauges) the accuracy of the calculated in situ stresses is ±1.2 MPa. The accuracy of displacements measure was way better than for the stresses, thus the displacements comparisons, presented in the preceding paragraph, seem to be more reliable and because of that were used for the purpose of the backfill stiffness properties calibration. From this reason the discussion about the cause of differences between measurements and numerical calculations has been done based on the registered displacements. Although the accuracy of stress measurements is not so good in relation to the values that were registered, which were relatively small and close to the device accuracy, it can be still concluded that there definitely is a correspondence between the in situ measurements and FEM estimations in the field of stress comparisons.

## 7. Conclusions and Final Remarks

Interdisciplinary and comprehensive non-destructive diagnostic tests of final bridge inspection and acceptance of a soil-steel bridge made of corrugated sheets, being the European span length record holder (25.74 m), done in the year 2017 and 2018, were described. Non-standard program of bridge loading was used during the inspection. On the one hand, classically, static loads were applied to the bridge by appropriate arrangement of trucks. On the other hand, the bridge response was studied under moving loads, to reduce permanent deformations when it was unloaded.

Because of the large dimension of the structure and also because its deformations under standard traffic loads are very small (a few mm only), sophisticated terrestrial laser scanning was done in order to measure short-term response of the bridge. Its accuracy was significantly increased by additional correction of results based on total station surveying and inductive sensors vertical displacement control. The resulting 3D deformation images of the steel shell sections, with the achieved precision (X, Y ~0.1 mm; Z ~0.01 mm; for the 2 mm × 2 mm grid of measurement points) and accuracy of the laser scanning ~0.3 mm, can be considered as a unique achievement. Such a high precision and accuracy of the geodetic measurement allowed to reliably identify bridge displacements, which under maximum loads were not more than 1.7 mm. Moreover, this image satisfies the conditions of the reference state in the case of structure monitoring, and possible periodic diagnostics during its life cycle.

A control of the structure geometry shape was done one year after the final inspection. In effect, maximum total displacement of the bridge, which occurred in one-year period, determined in terrestrial laser scanning equaled 7 mm. The scan accuracy in this case was ~2 mm. This is typical for structures of this type. Thus, we can say that the bridge experienced a change of its shape, which however does not affect its response and load bearing capacity.

A computational model of the bridge was built in order to verify the appropriateness of the design process. In situ short-term response measurements were compared with the numerical calculations resulting from FEM analyses. Calibration of the backfill elastic modulus based on the measured response of the bridge was done and the resulting accuracy of the displacements predictions was at the level of 95%. The model reflects the real response of the structure in the field of both quality and quantity of displacements and its strain. This confirms that the approach to modelling proposed in this paper was appropriate and that the bridge was correctly designed. In effect it can be used for the purpose of the bridge diagnostics or support interpretation of SHM data.

The bridge underwent all the required final acceptance tests and inspections and currently there are no doubts about its behavior during the designed lifetime. Moreover, its accurate periodic diagnostics is also possible.

## Figures and Tables

**Table 1:** Comparison of vertical displacements measured by means of total station and inductive sensors during S2 tests in the section 4-4.

Type of Measurement	Vertical Displacement (mm)		
	Point 2	Point 4	Point 6
Total station (*p* measuring point)	0.2	1.7	0.4
Inductive sensor (*u* measuring points)	0.07	1.59	0.28

**Table 2:** Yielding of steel rafter in all the considered local models.

Material	Elastic Modulus (MPa)	Poisson’s Ratio
Steel corrugated sheets	210,000	0.3
Soil (backfill)	170−230	0.2

**Table 3:** Comparison of the in situ measured and estimated by means of FEM extreme displacements.

Displacement	p4/2 (mm)	p4/4 (mm)
FEM value, backfill E = 170 MPa U_FEM_^E = 170^	1.91	2.09
FEM prediction, backfill E = 200 MPa U_FEM_^E = 200^	1.74	1.86
FEM prediction, backfill E = 230 MPa U_FEM_^E = 230^	1.53	1.68
In-situ, elastic value U_in-situ,el_	1.5	1.6
In-situ, total value U_in-situ,tot_	1.7	1.7

**Table 4:** Comparison of the in situ measured and estimated by means of FEM displacements in p4/4 measuring point during the moving load tests (M21–M26).

Test Denotation	FEM Value U_FEM_^E = 170^, Backfill E = 170 MPa (mm)	FEM Value U_FEM_^E = 200^, Backfill E = 200 MPa (mm)	FEM Value U_FEM_^E = 230^, Backfill E = 230 MPa (mm)	In-Situ Value U_in-situ_ (mm)
M21	−0.03	0.02	−0.02	0.10
M22	0.04	−0.04	0.05	0.20
M23	0.69	0.63	0.58	0.70
**M24**	**1.54**	**1.38**	**1.25**	**1.30**
M25	0.36	0.34	0.32	0.40
M26	−0.02	0.02	0.01	0.10

**Table 5:** Comparison of the in-situ measured and estimated by means of FEM (for the backfill, having E = 200 MPa) extreme stresses.

Stress	t2/4 (MPa)	t3/4 (MPa)	t4/4 (MPa)
FEM prediction S_FEM_, backfill E = 200 MPa	−6.21	−1.31	−6.52
In-situ, elastic value S_in-situ,el_	−5.74	−2.13	−6.24
In-situ, total value S_in-situ,tot_	−6.61	−2.50	−6.49

**Table 6:** Comparison of the in situ measured and estimated by means of FEM (for the backfill, having E = 200 MPa) stresses in t2/4 measuring point during the moving load tests (M21–M26).

Test Configuration	FEM Prediction S_FEM_ (MPa)	In-Situ Measure S_in-situ_ (MPa)
M21	−0.17	0.12
M22	−0.11	0.25
M23	−1.41	−1.12
**M24**	**−4.55**	**−3.99**
M25	−1.44	−1.12
M26	−0.14	0.37
